# A matter of you versus me? Experiences of control in a joint go/no-go task

**DOI:** 10.1007/s00426-017-0903-5

**Published:** 2017-08-23

**Authors:** Anouk van der Weiden, Roman Liepelt, Neeltje E. M. van Haren

**Affiliations:** 1 Social Health and Organizational Psychology, Utrecht University, Heidelberglaan 1, 3584 CS Utrecht, The Netherlands; 2 Brain Center Rudolf Magnus, University Medical Center Utrecht, Huispostnummer A.01.126, PO Box 85500, 3508 GA Utrecht, The Netherlands; 3 Department of Psychology, German Sport University Cologne, Am Sportpark Müngersdorf 6, 50933 Cologne, Germany; 4 Institute for Psychology, University of Muenster, Fliednerstrasse 21, 48149 Münster, Germany

## Abstract

When interacting with others, people represent their own as well as their interaction partners’ actions. Such joint action representation is essential for action coordination, but may also interfere with action control. We investigated how joint action representations affect experienced control over people’s own actions and their interaction partners’ actions. Participants performed a joint go/no-go task, which is commonly used to measure to what extent people represent their own actions in spatial reference to their interaction partner (e.g., as ‘left’ vs. ‘right’). After each second trial, participants indicated experienced control over their own action, their interaction partner’s action, or over action inhibition. Despite this frequent interruption of the go/no-go task, we found strong evidence for the spatial representation of joint actions. However, this joint action representation did not affect experiences of control. Possible explanations and implications of these findings are discussed.

## Introduction

In our everyday life, we regularly coordinate our actions with others, e.g., to divide labor and save energy, or to make use of each other’s expertise and accomplish more than we could on our own (Beckes & Coan, 2011; Coan, Schaefer, & Davidson, 2006; Schnall, Harber, Stefanucci, & Proffitt, 2008; Sebanz, Knoblich, & Prinz, 2003). However, the joint coordination of actions also introduces ambiguity in action control. That is, merely observing other people’s actions activates the same brain areas as when we perform the action ourselves (Carr, Iacoboni, Dubeau, Mazziotta, & Lenzi, 2003; Cochin, Barthelemy, Roux, & Martineau, 1999; Hari et al., 1998; Mukamel, Ekstrom, Kaplan, Iacoboni, & Fried, 2010). Hence, during action coordination, it may be hard to distinguish our own actions and their consequences from those of others (Brass, Ruby, & Spengler, 2009; Liepelt et al., 2016). If people integrate (and potentially confuse) their own and other people’s actions during joint action control, how then, do they experience control over jointly coordinated actions?

Over the past decade, research has revealed much on how people experience control over their own behavior in social isolation (Aarts, Custers, & Wegner, 2005; Frith, Blakemore, & Wolpert, 2000; Moore, Lagnado, Deal, & Haggard, 2009; van der Weiden, Aarts, & Ruys, 2013; Wegner & Wheatley, 1999). This research has demonstrated that experienced control crucially depends on how people represent their behavior (Aarts et al., 2012; Aarts, Custers, & Marien, 2009; David et al., 2006; Pacherie, 2008; Vallacher & Wegner, 1989; van der Weiden, Aarts, & Ruys, 2010; van der Weiden, Ruys, & Aarts, 2013). That is, people typically experience control over an action (e.g., playing piano) when its triggers (e.g., notes on the bass clef), performance (e.g., a left-hand movement), and consequences (e.g., low tones) match the prior internal representation of the action (e.g., when the action was represented in terms of notes on the bass clef, left-hand movements, and low tones; Moore et al., 2009; Morsella et al., 2009; Sidarus & Haggard, 2016; van der Weiden et al., 2010; van der Weiden, Aarts, & Ruys, 2011; Wenke, Fleming, & Haggard, 2010).

As actions are typically represented in terms of desired outcomes (i.e., in terms of overarching goals; Vallacher & Wegner, 1987; van der Weiden et al., 2010), people usually experience control when they attain their (conscious or unconscious) goals, even in the absence of actual control (Aarts et al., 2009, 2005; Preston & Newport, 2010; J. L. Preston, Ritter, & Wegner, 2011; van der Weiden, Ruys, et al., 2013). But also earlier action selection processes that take place before goal attainment play an important role, such that experienced control is strongest when a matching representation of the required action is pre-activated (i.e., in the absence of response conflict; Morsella et al., 2009; Sidarus & Haggard, 2016; Wenke et al., 2010). In one study that tested the role of action selection processes in experienced control, participants performed a Stroop task (Stroop, 1935) in which they had to name the font color of stimulus words (e.g., blue), while the stimulus words sometimes triggered a different naming response (e.g., when the word was “red”), inducing response conflict. After each trial, participants indicated to what extent they experienced control over their response. Results showed that participants experienced more control over their actions when a representation of the required response (e.g., blue) was triggered by a matching stimulus word (e.g., “blue” vs. “red”).

Such response selection processes become more complicated when coordinating multiple (e.g., left and right hand) actions simultaneously. That is, each action requires different planning and execution and may be associated with different triggers and with different expectations regarding its consequences (e.g., when playing piano, notes on the bass clef trigger left-hand actions that produce low tones, while notes on the treble clef trigger right-hand actions that produce high tones). In order to successfully distinguish and control multiple actions, people tend to represent (*or code*) their actions in reference to each other (e.g., as “bass,” “left,” “low” vs. “treble,” “right,” “high”; Craft & Simon, 1970; Elsner & Hommel, 2001; Hommel, 1993, 1996b; Simon, 1990), also referred to as *referential coding*. As such, people can represent (and experience control over) multiple actions at the same time. With regard to experienced control, one would expect the strongest experiences of control when one’s actions are compatible with these referential representations (e.g., when left-hand movements produce low rather than high tones).

Although experiences of control have been associated with social behavior and moral responsibility (Damen, van Baaren, Brass, Aarts, & Dijksterhuis, 2015; Frith, 2013; Moretto, Walsh, & Haggard, 2011; Ruys & Aarts, 2012), relatively little is known about how such processes affect people’s experiences of control when interacting with others. First evidence on experiences of control in joint action suggests that similar to individual action, people experience more control over jointly coordinated actions that lead to goal attainment (Dewey & Carr, 2013; Dewey, Pacherie, & Knoblich, 2014; van der Wel, Sebanz, & Knoblich, 2012). Yet, the question remains whether experiences of control are affected by the way people represent their own actions and those of their interaction partner.

In essence, the coordination of actions with others is not so different from the coordination of one’s own actions. Indeed, people also referentially code their own actions in reference to their interaction partner, which has repeatedly been demonstrated in a go/no-go task that is commonly used to measure referential coding (i.e., the Simon task). In a standard version of this task participants respond with different actions to different colored stimuli that pointed to the left, middle, or right of the computer screen. For example, participants are required to respond as fast as possible to green stimuli with a left button press and to red stimuli with a right button press. This typically results in a Simon effect (Craft & Simon, 1970; Simon, 1990), i.e., slowed reaction times to stimuli that are incompatible with the spatial referential coding of actions (e.g., responding with a *right* button press to a stimulus pointing to the *left*). Intriguingly, while there is typically no Simon effect when people have to respond to only one stimulus in an individual go/no-go version of the task (e.g., only responding to green stimuli with left button presses; Hommel, 1996a; Sebanz et al., 2003), the Simon effect is reinstated when another person or object is associated with the other stimulus (e.g., when one’s interaction partner responds to the red stimuli with right button presses; Sebanz et al., 2003). This reinstatement of the Simon effect suggests that just like representing their own (e.g., left and right hand) actions in reference to each other, people tend to represent their own actions in reference to other events in their direct environment, such as their interaction partner’s actions when jointly coordinating actions (Dolk et al., 2014; Dolk, Hommel, Prinz, & Liepelt, 2013; Sebanz et al., 2003; Tsai, Kuo, Hung, & Tzeng, 2008). To the extent that people represent their own as well as their interaction partner’s actions during action coordination, people may experience control over their own as well as others’ actions that are compatible with these representations.

Specifically, we expect people to experience more control over their own as well as their interaction partner’s actions when these actions (e.g., left key presses) are triggered by a compatible (e.g., left-presented) rather than an incompatible (e.g., right-presented) stimulus. However, as referential coding (Dittrich, Rothe, & Klauer, 2012; Liepelt, Wenke, Fischer, & Prinz, 2011; Vlainic, Liepelt, Colzato, Prinz, & Hommel, 2010) and neural activation (Kilner, Friston, & Frith, 2007; Mukamel et al., 2010; Veluw & Chance, 2014) are generally stronger for own compared to others’ action performance, we do expect people to experience more control over their own actions compared with their interaction partner’s actions. To test these hypotheses, we employed a joint go/no-go task in which we assessed experiences of control over (1) self-performed actions on go-trials, (2) other-performed actions on no-go trials, and 3) action inhibition on no-go trials.

## Methods

### Ethics statement

This study has been approved by the local ethics committee and has been performed in accordance with the ethical standards laid down in the 1964 Declaration of Helsinki. All participants gave their informed consent prior to their inclusion in the study.

### Participants and design

Twenty-seven undergraduate students of Utrecht University participated in this study for a small fee or course credits. The study had a 2 (spatial compatibility: incompatible vs. compatible) × 2 (action type: go vs. nogo) × 2 (experienced control question on no-go trials: action inhibition vs. other’s action) mixed design with spatial compatibility and action type as within-subject variables and the experienced control question on no-go trials as between-subjects variable.

### Experimental task and procedure

#### Joint go/no-go task

**Fig. 1:**
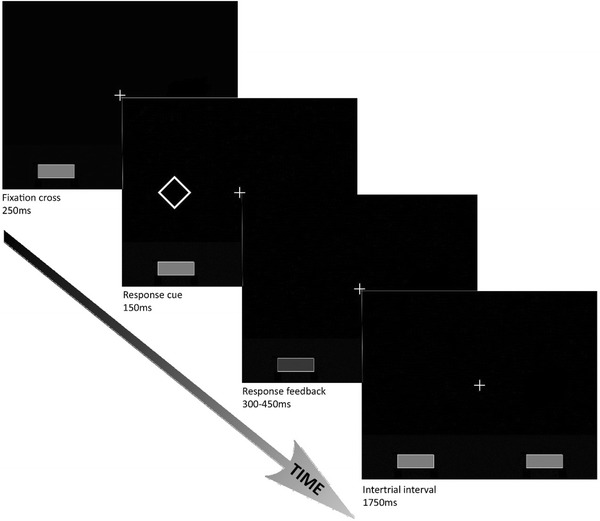
Schematic example of a compatible joint Simon trial in which stimulus location and response are compatible (*both left*)

Participants performed an adapted version of a well-documented joint go/no-go task, i.e., the joint Simon task (Liepelt et al., 2011; Sebanz et al., 2003). In this joint Simon task, participants responded to geometrical shapes (i.e., diamonds or squares) on the computer screen together with a virtual interaction partner whose actions were displayed on the lower left side of the screen (see Fig. 1). Participants were told that this virtual interaction partner was another participant who responded from a nearby computer cubicle, but in fact, the other’s actions were pre-programmed^1^. As such, the interaction partner’s action performance was stable across participants. Each actor was responsible for responding to only one of two shapes. For example, the participant had to respond to diamonds by pressing a right button, while the virtual interaction partner had to respond to squares by pressing a left button. The location of the displayed shape varied (left or right on the computer screen) to be compatible or incompatible with the participant’s location and button press (always on the right).

At the beginning of each trial, participants saw a fixation cross. After 250 ms, a diamond or square would appear left or right from the fixation cross for 150 ms. Depending on the shape, either the participant or the virtual interaction partner responded as fast as possible (reaction times ranged from 300 to 450 ms for the virtual interaction partner), while the other actor had to refrain from responding. Feedback was presented immediately after action performance for 300 ms. Specifically, the gray response button of either the interaction partner (left) or the participant (right) turned dark. In addition, for responses on no-go trials (false alarms) the message ‘FOUT’ (false) was presented, for non-responses on go-trials (misses) the message ‘TE LANGZAAM’ (too slow) was presented (1800 ms after stimulus onset), and for correct responses (hits and correct rejections) no explicit feedback was given (participants saw a fixation cross). To avoid confusion, no error feedback was presented for the interaction partner’s actions. Subsequently, the next trial started after a 1750 ms inter-trial interval.

The task consisted of 320 trials of which 160 were go-trials (participant had to respond) and 160 were no-go trials (virtual interaction partner responded). In 80 of these trials, the location of the presented shape (e.g., diamond, right) was compatible with participants’ location and button press (i.e., right). In another 80 trials, the location of the presented shape (e.g., diamond, left) was incompatible with participants’ location and button press (i.e., right). In order to control for potential sequential effects, each of eight possible trial combinations (e.g., compatible go-trial followed by an incompatible no-go trial) was presented 40 times. Trial sequences were randomly presented in 5 blocks of 64 trials with short 10-s breaks in between.

#### Experiences of control

Following each second trial in a trial sequence, participants were asked to indicate (on a scale from 0 to 9) the extent to which they felt control on the preceding go or no-go trial. As such, experienced control was rated on 160 trials, of which 80 go-trials and 80 no-go trials. For go-trials, all participants were asked “to what extent did you feel in control over reacting as fast as possible to the [*square or diamond*]?”. For the no-go trials, the first 16 participants rated experienced control over the interaction partner’s actions (“to what extent did you feel in control over the other person’s reaction to the [*square or diamond*]?”), while the other 11 participants rated experienced control over not acting (action inhibition; “to what extent did you feel in control over NOT reacting to the [*square or diamond*]?”).

## Results

Erroneous responses as well as responses below 100 ms or above 1000 ms (1.46% of our data) were excluded from the analyses (Ratcliff, 1993). One participant was excluded for making too many errors (9.06%; >3 SD above the average of 1.96%). As response cue (diamond vs, square) and the type of question asked on no-go trials (control over action inhibition vs. other’s action) did not interact with spatial compatibility (all *p*’s > 0.05) in any of the analyses reported below, we computed mean RT’s (in ms) on compatible and incompatible trials, collapsing across response cue and no-go question. Because the scales on which reaction times and experienced control were measured are incompatible, we performed separate repeated measures ANOVA’s.

### Confirmatory analyses

#### Reaction times: frequentist analysis

First, we subjected participants’ mean RT’s to a repeated measures ANOVA with spatial compatibility (incompatible vs. compatible) as within-participants variable. This analysis revealed the expected main effect of spatial compatibility, *F*(1,25) = 52.46, *p* < 0.001, *η*_*p*_^2^ = 0.68. Participants were on average 16.65 ms faster to respond to compatible (*M* = 402, SD 46.1) compared with incompatible (*M* = 418.6, SD 46.5) stimuli.

#### Reaction times: bayesian analysis

We then tested the same design using Bayesian statistics in JASP (JASP Team, 2016). This analysis confirmed that there is strong support for the presence of a compatibility effect on reaction times [bayesian factor (BF_10_/BF_M_/BF_inclusion_) = 52,085.93, meaning that the data are estimated to be 52,085.93 times more likely to occur under the model that includes compatibility as a factor than under the null model]. Note that, a BF of 3 is approximately equal to a *p* value of 0.05.

#### Experiences of control: frequentist analysis

**Fig. 2:**
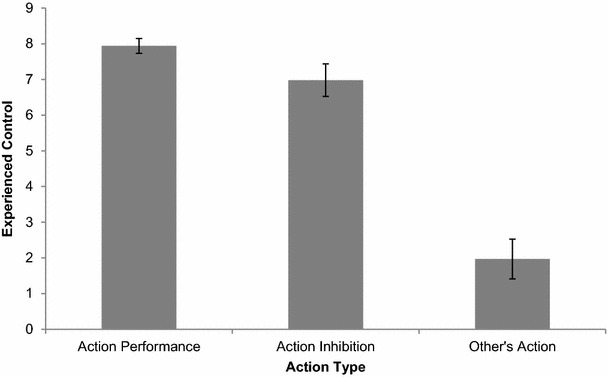
Experienced control as a function of stimulus–response compatibility and action type (own action, action inhibition, other’s action). *Error bars* represent standard errors of the means

Next, we subjected participants’ mean experiences of control over their own actions (on Go-trials), action inhibition (for participants who rated experienced control over action inhibition on NoGo-trials), and other’s actions (for participants who rated experienced control over their interaction partner’s action on NoGo-trials) to a repeated measures ANOVA with spatial compatibility (incompatible vs. compatible) and TrialType (Go vs. NoGo) as within-subjects variables and NoGoQuestion (Inhibition vs. Other’s action) as a between-subjects variable. In contrast to our prediction, there was neither a main effect of spatial compatibility on experienced control (*F*(1,24) = 3.31, *p* = 0.08, *η*_*p*_^2^ = 0.12), nor an interaction with the type of action for which experienced control was rated (action performance vs. action inhibition vs. other’s action; *F* < 1). Figure 2 represents mean experiences of control for each action type (action performance, action inhibition, and other’s actions).

However, there was a main effect of TrialType (*F*(1,24) = 61.93, *p* < 0.001, *η*_*p*_^2^ = 0.72), such that experienced control was higher on go-trials compared to no-go trials. This go/no-go effect was further qualified by an interaction with the type of action for which control was rated on no-go trials (NoGoQuestion: action inhibition vs. other’s actions; *F*(1,24) = 37.37, *p* < 0.001, *η*_*p*_^2^ = 0.61). To gain further insight in this interaction effect, and to test our specific predictions, we performed separate within-subjects comparisons between experienced control over go-trials versus no-go-trials, as a function of whether experienced control was rated for action inhibition or the interaction partner’s actions. Results suggested that experienced control over action performance (go-trials; *M* = 7.79, SD 0.86) was higher compared with *action inhibition* (no-go trials; *M* = 6.98, SD 1.51), *F*(1,10) = 14.18, *p* = 0.004, *η*_*p*_^2^ = 0.59. Also, experienced control was higher for self-performed actions (go-trials; *M* = 7.99, SD 1.20) compared with others’ actions (no-go trials; *M* = 1.97, SD 2.16), *F*(1,14) = 68.28, *p* < 0.001, *η*_*p*_^2^ = 0.83. Between-subjects comparisons between experienced control over action inhibition versus others’ actions revealed that participants experienced more control over their own action inhibition compared with their interaction partner’s actions, *F*(1,25) = 43.42, *p* < 0.001, *η*_*p*_^2^ = 0.64.

#### Experiences of control: bayesian analysis

We again tested the same design using Bayesian statistics in JASP (JASP Team, 2016). This design included three factors (Compatibility, TrialType, & NoGoQuestion), and thus gives us 19 models (including *H*_0_) that can be compared. Because it is risky to base conclusions on a comparison involving only a small subset of models, we decided to apply bayesian model averaging (BMA) in order to retain model selection uncertainty. In line with this decision, we will report BF_inclusion_ values, which indicate the likelihood of the data under models that include a certain factor compared to models that do not include this factor. We also manually set the number of samples to 500,000 to reduce the Monte Carlo sampling error. Averaged across all candidate models, the data strongly support the inclusion of NoGoQuestion (inhibition vs. other’s action; BF_inclusion_ = ∞) and TrialType (go vs. no-go; BF_inclusion_ = ∞), as well as their interaction (BF_inclusion_ = 3.753E + 14). The BF_inclusion_ for compatibility was 0.127, indicating that the data are 7.874 (1/0.127) times more likely under models that do *not* include Compatibility as a factor compared with models that *do* include Compatibility as a factor.

### Exploratory multilevel analyses on performance and experienced control

Although we did not find an effect of compatibility on experienced control, it would be interesting to test whether experienced control was related to actual control as indicated by accuracy and reaction time. We did not have specific hypotheses with regard to this relation, as previous research on this matter often shows no relation between action performance and experienced control (e.g., Chambon & Haggard, 2012; van der Weiden, Ruys, et al., 2013; Wenke et al., 2010).

#### Experienced control as a function of accuracy

Using a linear mixed models multilevel analysis (with accuracy person-mean centered), we found a significant association between accuracy and experienced control, such that experienced control is higher on correct (vs. incorrect) trials *β* = 1.840, 95% CI [0.841–2.839], *t*(21.70) = 3.822, *p* < 0.001. Furthermore, the strength of this association differs slightly per person, *β* = 4.034, 95% CI [1.926–8.449], Wald *Z* = 2.651, *p* = 0.01).

#### Experienced control as a function of reaction time

A second linear mixed models multilevel analysis (with reaction time person-mean centered) indicated that there is also a small but significant association between reaction time and experienced control, such that experiences of agency are higher when reaction times are faster, *β* = −0.003, 95% CI [−0.004 to −0.002], *t*(26.50) = −5.16, *p* < 0.001. Again, the strength of this association differs slightly per person, *β* = 6.27E−6, 95% CI [3.21E−6 to 1.22E−5], Wald *Z* = 2.93, *p* = 0.003.

### Exploratory trial order and sequential reaction time analyses

This is the first time the spatial compatibility effect has been studied in a joint Simon task that is regularly interrupted by self-report questions, creating short two-trial sequences. As it is not uncommon for researchers to exclude the first trial(s) of the joint Simon task from their analyses (e.g., Liepelt, Wenke, & Fischer, 2013; Liepelt et al., 2011; Notebaert, Gevers, Verbruggen, & Liefooghe, 2006; Verbruggen, Notebaert, Liefooghe, & Vandierendonck, 2006), we were curious whether the spatial compatibility effect only occurs on later trials when participants are in the flow of the task, or whether we can already detect joint Simon effects on first trials.

Furthermore, previous research has shown that the spatial compatibility effect in the joint Simon task is affected by the preceding trial type (compatible vs. incompatible), such that the joint Simon effect is stronger after compatible compared with incompatible trials (Akçay & Hazeltine, 2007; Fischer, Dreisbach, & Goschke, 2008; Hommel, Proctor, & Vu, 2004; Liepelt et al., 2011; Notebaert, Soetens, & Melis, 2001; Stürmer, Leuthold, Soetens, Schröter, & Sommer, 2002; Wühr & Ansorge, 2005). According to the feature-integration account, this sequential modulation of the (social) Simon effect occurs because of additive effects of compatibility and repetition of stimulus and response features (Hommel et al., 2004; Liepelt et al., 2011). That is, relatively fast reaction times on compatible trials are further sped up by the repetition of the same stimulus–response combination, while relatively slow reaction times on incompatible trials are further slowed down by a change in the stimulus–response combination after compatible trials. In contrast, after incompatible trials, the relatively fast reaction times on compatible trials are slowed down by a change in the stimulus–response combination, while relatively slow reaction times on incompatible trials are sped up by a repetition of the same stimulus–response combination. Because we tested each possible sequential trial combination, we were able to assess such sequential effects on reaction times as well as on experiences of control.

#### Trial order effects on reaction times: frequentist analysis

To test whether the spatial compatibility effect differed for first trials versus second trials in the trial sequences, we performed a repeated measures ANOVA with trial order (first vs. second trial in a sequence) and compatibility (incompatible vs. compatible) as within-subjects variables. Results again showed the main effect of compatibility, *F*(1,25) = 59.18, *p* < 0.001, *η*_*p*_^2^ = 0.70. There was also a main effect of trial order, such that participants were slower to respond on first trials (*M* = 428.3, SD 50.7) than on second trials (*M* = 377, SD 44.1), *F*(1,25) = 152.77, *p* < 0.001, *η*_*p*_^2^ = 0.86. Although the spatial compatibility effect on first trials (21.3 ms) appears to be larger than on second trials (12.9 ms), the interaction between compatibility and trial order did not reach statistical significance, *F*(1,25) = 3.81, *p* = 0.06, *η*_*p*_^2^ = 0.13.

#### Trial order effects on reaction times: bayesian analysis

To corroborate these findings, we again ran a Bayesian analysis on the same design. In line with the frequentist analysis, this analysis indicated that the data most strongly support a model including both main effects (compatibility and trial order; BF_M_ = 6.639), to the extent that this model is 6.6 times more likely than the alternative models (i.e., null model, one-factor models, and full model). The support for including the interaction term is inconclusive (BF_inclusion_ = 2.410).

#### Sequential reaction time analyses: frequentist analysis

**Fig. 3:**
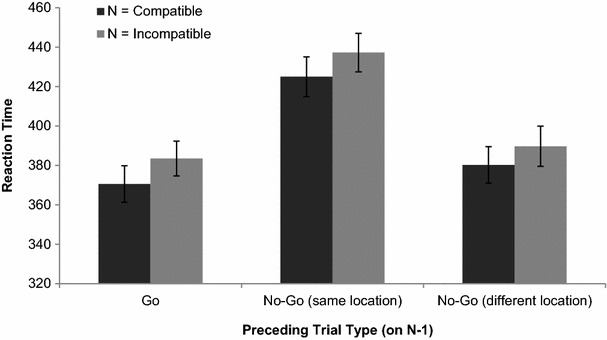
Reaction times on go-trials as a function of preceding trial type (go-trial, no-go trial with the same stimulus location, no-go trial with a different stimulus location) and compatibility on *N*. *Error bars* represent standard errors of the means

Next, we tested whether the spatial compatibility effect (on trial *N*) depended on the preceding trial type (*N* − 1). For this purpose, we performed a repeated measures ANOVA with compatibility on *N* (incompatible vs. compatible), trial type on *N* − 1 (incompatible vs. compatible vs. no-go), and planned contrasts between go (compatible and incompatible) vs. no-go as preceding trial type. This analysis revealed no main or interaction effects of compatibility on *N* − 1 (all *F*’s < 2.01, all *p*’s > 0.16). There was a main effect of whether the preceding trial was a go versus no-go trial on reaction times, such that reaction times were slower after no-go trials (*M* = 408,1, SD 47,7) compared with go-trials (*M* = 377, SD 46.1), *F*(1,25) = 52.48, *p* < 0.001, *η*_*p*_^2^ = 0.68. However, this effect did not interact with spatial compatibility on *N* (*F* < 1). Follow-up analyses indicated that reaction times were especially slowed down after no-go trials when a go-stimulus was presented on the same (vs. different) location as the no-go stimulus in the preceding trial (*M* = 431.1, SD 48.7 vs. *M* = 385, SD 47.2), *F*(1,25) = 109.42, *p* < 0.001, *η*_*p*_^2^ = 0.81 (see also Fig. 3).

#### Sequential reaction time analyses: bayesian analysis

A Bayesian analysis of the same design converged with the frequentist analysis, such that our data provided the strongest evidence for a model including both main effects of preceding trial type and compatibility on *N* (BF_M_ = 15.580). That is, reaction times were faster on compatible versus incompatible trials, and after go versus no-go trials. Evidence for an interaction between preceding trial type and compatibility on *N* was inconclusive (BF_inclusion_ = 1.004). The follow-up analysis again indicated strong evidence for larger decreases in reaction times after no-go trials when a go-stimulus was presented on the same (vs. different) location as the no-go stimulus in the preceding trial (BF_10_/BF_M_/BF_inclusion_ = 2.581E+7).

## Conclusion and discussion

In the present study, we investigated whether the internal representation of an interaction partner’s actions would lead to vicarious experiences of control. Although participants did represent their actions in reference to their interaction partner (as demonstrated by a strong main effect of spatial compatibility on reaction times), this joint action representation did not predict experiences of control over their own or their interaction partner’s actions. In fact, Bayesian analyses indicated that our data provided evidence for a null-effect of compatibility on experienced control. This null-finding appears to be inconsistent with research suggesting that experienced control over self-produced actions is strongest when action selection is smooth (i.e., on compatible compared with incompatible trials; Morsella et al., 2009; Sidarus & Haggard, 2016; Wenke et al., 2010).

Possibly, we found no effects of compatibility on experienced control because the difference between spatially compatible and incompatible trials was too subtle. That is, in contrast to a recent study by Sidarus (2016), the source of the response conflict in our study is implicit. Furthermore, although we found a strong compatibility effect in our study, the difference in reaction times for compatible and incompatible trials was only 16.65 ms, or even 12.92 ms if you only consider the second trials over which experienced control was assessed. Yet, reaction times in the studies by Morsella (2009; non-verbal condition) and Wenke (2010) differed by 133.4 and 49.9 ms between compatible and incompatible trials, respectively. Hence, even though participants may have been unaware of the source of the response conflict (e.g., incompatible action primes), they may have noticed the resulting differences in their own reaction times and used this information to base their experience of control on. Reaction time differences may thus affect experienced control only if they can be consciously detected by the actor. Conscious detection may, however, be especially relevant when explicitly assessing experienced control. A remaining question for future research is whether people do experience more control on an implicit level as a result of representing one’s own actions in reference to their interaction partner, and whether such an implicit sense of control affects behavior regulation (Steinhauser & Kiesel, 2011), (observational) learning, or feelings of responsibility (Frith, 2013; Haggard & Tsakiris, 2009; Moretto et al., 2011) for jointly produced actions and outcomes.

Further exploratory analyses did not reveal the typical sequential modulation of the joint Simon effect (i.e., the compatibility effect being larger after compatible versus incompatible trials; Liepelt et al., 2011) in our adapted version of the joint Simon task, which was regularly interrupted by a question about experienced control following each second trial. It could be that the facilitating effects of stimulus–response repetition are attenuated by the relatively large performance improvements on each second trial in a sequence. Such performance improvements are usually less pronounced as reaction times and sequential effects are typically measured over longer trial sequences, sometimes even excluding performance on the first trial from analyses (e.g., Liepelt et al., 2013, 2011; Notebaert et al., 2006; Verbruggen et al., 2006). More importantly, the presence of a joint Simon effect in the absence of the typical sequential modulation supports the assumption of the feature-integration account that the (social) Simon effect is independent from the integration processes that produce the sequential effects (Hommel et al., 2004).

It is worth noting that we were able to reliably detect the joint Simon effect even when interrupting the task every other trial with introspective questions. Although reaction times were slower on first trials compared to second trials, the spatial compatibility effect was present on first as well as second trials within a sequence. This opens new possibilities for assessing the consequences of joint action representations within the context of the (joint) Simon task. For example, it might be possible to add implicit measures of experienced control (e.g., such as temporal binding or sensory attenuation; see Hughes, Desantis, & Waszak, 2013) that may be more susceptible to subtle response selection processes. Also, it might be interesting to gain more insight in when and how people learn from the observation of other people’s actions, e.g., by measuring memory for self-performed versus observed reactions to compatible versus incompatible stimuli on a trial level.

In conclusion, in line with the notion that referential coding (Dittrich et al., 2012; Liepelt et al., 2011; Vlainic et al., 2010) and neural activation (Kilner et al., 2007; Mukamel et al., 2010; Veluw & Chance, 2014) are generally stronger for own compared to others’ action performance, the present findings indicate that experienced control is higher for self-produced versus other-produced actions. In that sense, experienced control is a matter of you versus me. However, the present study further suggests that the ability to discriminate one’s own and others’ actions by means of spatial coding does not affect explicit experiences of control. Furthermore, we showed that the joint Simon effect is independent from the integration processes that produce typical sequential effects, and that the joint Simon effect remains intact despite frequent interruptions by additional measurements (i.e., introspective questions on experienced control). As such, the present research opens new avenues for future research on the downstream consequences of joint action representation.

## Footnotes

1 As part of our debriefing, we asked participants to what extent they were aware of their co-actor during the task (which implies their belief in the presence of a co-actor; scored on a scale from 0 to 9). Most participants were to some extent aware of their co-actor during the task (*M* = 4.58, SD 2.30). Only one participant indicated to have been entirely unaware of the co-actor during the task. Exclusion of this participant did not change our findings.
